# Effect of Solvent Presoaking of FDM-Printed Conductive PLA Current Collectors in 3D-Printed Carbon Supercapacitors

**DOI:** 10.1021/acsaenm.4c00716

**Published:** 2025-03-01

**Authors:** Matthew Ferguson, Vladimir Egorov, Yan Zhang, Umair Gulzar, Colm O’Dwyer

**Affiliations:** † School of Chemistry, University College Cork, Cork T12 YN60, Ireland; ‡ AMBER@CRANN, Trinity College Dublin, Dublin 2 D02 W9K7, Ireland; § Environmental Research Institute, University College Cork, Lee Road, Cork T23 XE10, Ireland

**Keywords:** 3D printing, supercapacitor, PLA, energy storage, electrochemistry, additive manufacturing

## Abstract

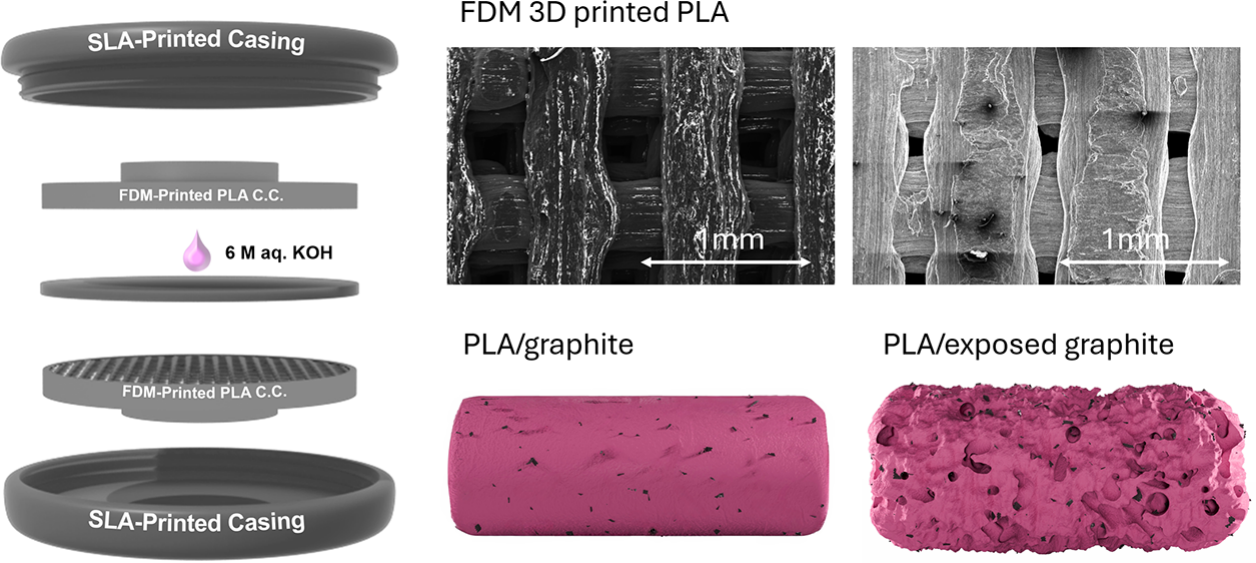

The electrochemical response of symmetric carbon-based supercapacitor devices made using two 3D-printing techniques, Vat-P (vat polymerization) and FDM (fused deposition modeling), shows how the printing method dominates the overall cell response. Despite possessing excellent cycle life, the conductive poly(lactic acid) (PLA) FDM printed current collectors suffer from relatively high resistance and suppressed capacitance linked to current collector material resistivity. Here, we examine in situ methods to influence the interfacial conductivity of the FDM current collectors by surface modification. Both dimethylformamide (DMF) and aqueous potassium hydroxide (KOH) treatments are investigated to compare solvent decomposition and electrolyte presoaking for this purpose. Using a single-walled carbon nanotube and graphene nanoplatelet carbon composite slurry on FDM current collectors in Vat-P 3D-printed cell casings, the supercapacitor cells show that the DMF treatment method has worse capacitance but better retention over 1 million cycles compared to the untreated FDM current collector cells. Pretreatment in a solution of 6 M aqueous KOH, identical to the cell electrolyte, markedly improves the effective current collector conductivity and interface with the active material, with a five-fold improvement in capacitance at the expense of less cycling stability. This is possible because the KOH treatment provides a 10-fold reduction in the FDM current collector resistance, which correlates with the improved cyclic voltammetric response. Galvanostatic charge–discharge tests reveal a deteriorated long-term cycling stability and rate capability despite better interfacial conductivity with the active material. In-situ presoaking that allows a degree of depolymerization at the surface relieves the conductive additive within the PLA to improve electrochemical interfacial activity and identifies the trade-off between improved capacitance and long-term cycling stability for common electrolytes in PLA-based 3D printed aqueous supercapacitors.

## Introduction

Additive manufacturing (more commonly known as 3D printing) has seen an explosion in popularity in recent years.^1−9^ Many areas of science are now extensively using 3D-printing in their research; biological engineering,^10−12^ healthcare,^13,14^ and even food sciences.^15,16^ The field of energy storage devices (more specifically, supercapacitors) is no exception; stereolithography (SLA), also generally known as vat polymerization (Vat-P),^17^ fused deposition modeling (FDM),^18,19^ direct ink writing (DIW),^20,21^ and other additive manufacturing methods^7,8^ have all been used for printing working symmetric carbon-based supercapacitors. Taking increasingly important environmental concerns into consideration, some researchers have used recycled paper, recycled conductive polylactic acid (PLA), and other carbon waste materials and sustainable waste biomass materials for making their 3D-printed electrodes.^22−25^ Some studies have designed a manufacturing process that results in a functional supercapacitor device made using only 3D printing,^18,26^ although most (including this work) require some level of manual assembly. The optimal, end-stage outcome of these supercapacitor studies is a fully automated additive manufacturing process, where all supercapacitor cell components are 3D-printed into a working device with minimal external assembly or processing required. Ideally, previously mentioned environmental concerns will be met, where raw materials are obtained from renewable and/or waste biomass sources.^27−29^

Our previous work involving 3D-printed supercapacitor devices details our findings for FDM-printed conductive PLA current collector cells.^30^ Despite possessing exceptional long-term cycling stability, the poor electronic conductivity of the FDM current collectors limited other electrochemical responses, resulting in nonideal cyclic voltammetry responses and large ohmic drops in galvanostatic charge–discharge tests.^31,32^ As outlined in other studies, the as-printed PLA material lacks the high conductivity necessary to act as an effective electrode/current collector.^6^ High electrical conductivity is essential for effective energy storage devices to ensure efficient charge transport within the active material slurry and its interface with the 3D-printed PLA current collector. As such, a postprinting treatment method is required to enhance the material’s electronic conductivity and the electrochemical performances of electrodes and current collectors made of this material. One such method is thermal treatment via carbonization, which both removes the insulating PLA and retains the 3D-printed structure. Redondo et al. tested the effect of carbonization temperatures on the morphology and electrochemical performances of 3D-printed conductive PLA electrodes.^33^ Though carbonization at 300 °C removed the maximum insulating PLA from the material, the same process at 500 °C achieved the largest improvement in capacitance and electrochemical performance. Other studies show similar improvements in electrochemical performance, while simultaneously retaining the detail and intricacy of 3D-printed electrodes.^34^

Chemical activation methods have been used extensively to improve the electronic conductivity of PLA-carbon composite materials. Gusmão et al. investigated the chemical activation ability of several common polar protic and polar aprotic solvents on 3D-printed conductive PLA electrodes.^35^ Polar aprotic solvents (such as acetone and DMF) were found to be far more effective at chemical activation than polar protic solvents (DI water, MeOH, EtOH), with the DMF-activated conductive PLA electrode outperforming all other conductive PLA electrodes in the study when it came to supercapacitor response. Dimethylformamide (DMF) has been used in other studies for the solvent activation of polymer/graphene filaments used in 3D-printed supercapacitors.^1,36,37^ The solvent causes the insulating PLA polymer to swell, exposing more of the filament’s conductive carbon material.^35^ Aqueous KOH solution (and other basic solutions, such as NaOH) have been used for chemical activation of carbon-based materials^38−40^ and alkaline etching of PLA and other polymer filaments.^41^ As an aliphatic polyester, PLA is susceptible to saponification, whereby it can be selectively removed using either an alkaline solution^42^ or the electrolysis of water,^43^ which differs from the physical swelling and roughening of PLA composites that occurs when treated with DMF or other polar protic solvents.

Chemical treatment is a relatively straightforward activation method that can be undertaken at room temperature, ideal for temperature-sensitive conductive PLA current collectors. DMF and aqueous KOH were used for these treatments. Since the 3D printed cell uses aqueous KOH-based electrolytes, we investigated electrolyte presoaking with the electrolyte as one of the chemical pretreatments and presented a detailed examination of the different behaviors and electrochemical performances of chemically treated conductive PLA FDM printed current collectors incorporated into 3D-printed symmetric carbon supercapacitor cells. These behaviors and performances are directly compared to our previous work involving FDM printed conductive PLA current collectors.^30^ This study shows that electrolyte presoaking is effective for FDM materials such as PLA in supercapacitors under voltammetric conditions. It also demonstrates that long-term cycling is relatively stable, albeit with a very low capacitance, since we set out to see how the underlying PLA is depolymerized and its internal graphitic content electrically interfaces with the added active carbon slurry. At reasonably high fixed currents, we see the limitation of resistive PLA in spite of improved surface conductivity, with severely suppressed capacitance during cycling as a result of a failure mode in PLA exposed to electrolyte over longer periods. Changes to the PLA that improve interfacial conductivity are maintained during operation within symmetric carbon supercapacitor cells, and pretreatment in the working electrolyte is a simple and effective way of modifying the conductive PLA, but under galvanostatic conditions, it limits long-term performance due to in situ degradation of the PLA composite.

## Experimental Section

### Printing and Processing of 3D-Printed Supercapacitor Components

A combination of Vat-P and FDM 3D printing was implemented in this study. The outer casings of the supercapacitors were printed using Vat-P, and the current collectors were printed using FDM. The standard batch of cells printed consisted of six full supercapacitor cells, with 12 FDM current collectors and 12 Vat-P half-casings printed per batch.

The model of the Vat-P printer used in this work is the FormLabs Form 2, and the model of the FDM printer is the MakerBot Replicator 2X. These two printers have the highest print resolution of 0.025 mm (© FormLabs 2023)^44^ and 0.1 mm (© FutureMakers)^45^ respectively, making the Vat-P printer a better choice for intricate, complex parts. However, these resolutions are reflected in the printing times. The Vat-P printing time in this work was 4 h (for 12 half-casings), while the FDM printing time was 1.5 h (for 25 current collectors). In terms of postprocessing, a time-consuming procedure of washing, curing, removing supports, and filing the printed parts to smoothness must be completed for the Vat-P printed components (up to 1.5 h in duration per batch of printed components). No postprocessing is required for FDM-printed parts. The raw resin used for all Vat-P printing is FormLabs V4 Clear Resin (© FormLabs 2024).^46^ ProtoPasta Conductive PLA thermoplastic (500 g, 1.75 mm diameter spool) is used for all FDM printing (© ProtoPlant 2024).^47^

For the Vat-P outer casings of the cells, the components were designed using CAD software, saved as a.form file, and uploaded to the Form 2 Formlabs Vat-P printer (© Formlabs, Somerville, Massachusetts, USA) via the Formlabs app. The resin used for all Vat-P components was Preform Clear V4 resin (© Formlabs, Somerville, Massachusetts, USA), at a resolution of 0.025 mm (the highest resolution on this printer). Once uploaded, the components were printed. The printing process (for 12 half-casings) took 4 h at a resolution of 0.025 mm. Once printed, the parts were removed from the building platform using a scraper and placed into an isopropyl alcohol (IPA) bath, where they were washed for 10 min to remove any residual liquid resin. Once washed, they were removed from the bath and dried. The parts were then placed into the curing machine for 15 min at a temperature of 60 °C (time and temperature are specific to each resin type). The Vat-P components were printed with supports, which prevent deformation during the printing process.^48,49^ The supports were broken off by hand or using a wire cutter. Any small bumps left by the supports were filed away by using sandpaper.

The FDM current collectors were printed by using a MakerBot Replicator 2X FDM printer (© UltiMaker, Utrecht, Netherlands). The material used for these current collectors was ProtoPasta Conductive PLA (© ProtoPlant, Vancouver, Washington, USA) filament (PLA plastic loaded with graphene for conductivity). Once the current collectors were designed using CAD, the design was uploaded to the MakerBot app, where the settings and layout of the printed components were finalized. The printer platform was set to a temperature of 115 °C and the extruder to a temperature of 230 °C (ideal temperature for melting the PLA filament without burning). Printing a batch of 25 FDM current collectors took ∼1.5 h at a resolution of 0.1 mm (the highest resolution possible for this FDM printer type and model). Once printing was completed, any defective current collectors were recycled (defects are far more common with this 3D-printing method, relative to Vat-P 3D-printing). No postprocessing was needed for this current collector type.

### Surface Modification of FDM Current Collectors

Three different chemical treatments were utilized in this study, with the aim of improving the conductivity of the FDM current collectors and the electrochemical performance of the supercapacitor cells they are used in. n,n-Dimethylformamide (DMF) (>99.8%), and 6 M aqueous potassium hydroxide (KOH) solution were used as the reagents for the treatments. The KOH solution was using 33.66 g of KOH (≥85%, pellets) in 100 mL of water. This solution is also used as the electrolyte in all supercapacitor cells in this study. Both the n,n-DMF and KOH pellets were purchased from Sigma-Aldrich (Gillingham, UK).

The first chemical treatment (DMF (short)) used n,n-DMF as the reagent. Twelve untreated FDM current collectors were placed in individual small polystyrene vials. A volume of 1.5 mL of DMF was added to each of the vials using a 1 mL syringe, completely submerging the current collectors and covering their surfaces in the reagent. The current collectors were left to sit in the reagent for 10 min, following which the vials were sonicated for 5 min using a Cole-Palmer 8891 Ultrasonic Cleaner (© 2023 Cole-Parmer Instrument Company, LLC), with the water bath at 20 °C and a frequency output of 42 kHz. After this, the current collectors were rinsed in fresh DMF, patted dry with tissue paper, and left in the fume hood overnight to completely dry in the air. Once completely dried, the current collectors were weighed and numbered.

The second chemical treatment (DMF (Long)) also used n,n-DMF as the reagent. Twelve untreated FDM current collectors were placed in individual small polystyrene vials. A volume of 1.5 mL of DMF was added to each of the vials using a 1 mL syringe, completely submerging the current collectors, and covering their surfaces in the reagent. This time, the current collectors were left to sit in the reagent for 72 h, following which the vials were sonicated for 5 min (using the same ultrasonic cleaner and settings as the DMF (Short) procedure above). After this, the current collectors were rinsed in fresh DMF, then patted dry with tissue paper, and left in the fume hood overnight to completely dry in the air. Once completely dried, the current collectors were weighed and numbered.

The final chemical treatment (KOH (Long)) used a 6 M aqueous KOH solution as the reagent. Twelve untreated FDM current collectors were placed in individual small polystyrene vials. A volume of 1.5 mL of KOH was added to each of the vials using a 1 mL syringe, completely submerging the current collectors and covering their surfaces in the reagent. The current collectors were left to sit in the reagent for 72 h, following which the vials were sonicated for 5 min (using the same ultrasonic cleaner and settings as the other two chemical treatment procedures). After this, the current collectors were rinsed in fresh KOH, and then rinsed in deionized water. After being patted dry with tissue paper, the current collectors were left in the fume hood to completely air-dry overnight. Once completely dried, the current collectors were weighed and numbered.

Four groups of FDM current collectors were studied in this work (untreated, DMF-treated short, DMF-treated long, and KOH-treated (long)). Each group contained 12 current collectors, enough to make 6 supercapacitor cells. The Untreated current collectors were used in the supercapacitor cells as printed without any chemical treatment. After their respective chemical treatments (or lack thereof, for the untreated current collectors), all current collectors were loaded with active materials and assembled into supercapacitor cells. The current collectors were completely submerged in their respective chemicals so that the entire surface of the current collector (and the wetted interior) would be treated. This was done instead of submerging only one face of the current collector (leaving the outer face untreated), as the improved electronic conductivity of the treated face could be limited by the poorer electronic conductivity of the untreated face. None of the current collectors in this study underwent more than one chemical treatment. Additionally, both current collectors within a given supercapacitor cell underwent the same treatment or lack thereof.

### Carbon Slurry Preparation and Loading onto Current Collectors

All materials in the carbon slurries were used as received with no purification steps. Single-walled carbon nanotubes (SWNTs, ≥ 99% carbon, ≥ 93% SWNT) and graphene nanoplatelets (GNPs) were purchased from Sigma-Aldrich (Gillingham, UK).

The active electrode material in these experiments was a simple carbon slurry consisting of a 50:50 mixture by mass of single-walled carbon nanotubes (SWNT) and graphene nanoplatelets (GNP). Each slurry batch consisted of 0.067 g of carbon materials (0.0335 g of SWNT and 0.0335 g of GNP) mixed in 5 mL of ethanol (EtOH). The slurry materials were weighed via weigh boats and then carefully added to a small sample vial. A volume of 5 mL of EtOH was added to the sample vial (while washing down any solid materials on the inner walls of the vial) using a 5 mL syringe. After adding a magnetic stirring bar, the vial was stopped and accurately labeled (date, slurry contents, masses, experiment number). The top of the vial was wrapped in Bemis Parafilm “M” laboratory film to further seal the vial and prevent any EtOH from evaporating out of the mixture during the stirring process. The vial was placed on a stirring tray, and the slurry was left to mix and homogenize overnight.

After homogenization, a 1 mL syringe was used to measure and place 50 μL of slurry (0.05 mL mark on the syringe) onto the center of each preweighed and numbered current collector. The tip of the syringe was used to spread the slurry across the current collector surface as much as possible (while keeping the slurry as one connected “island”). A sample of each slurry batch was also placed onto glass slides for Raman spectroscopy analysis. The current collectors were left to air-dry overnight. Once dried, the current collectors were reweighed and their mass loadings were calculated and recorded.

### Assembling Supercapacitor Cells

We used 70 mm Whatman Glass Microfiber Filters as the separators for these cells. A hole puncher was used to obtain 16 mm circular separators from the larger 70 mm filters. The loaded current collectors were paired and placed within their half-casing. A separator was placed on one of the current collectors per pair.

A 6 M aqueous KOH solution was used as the electrolyte for all cells in this work. In the fume hood, five drops of electrolyte were added to the center of each separator. The two halves of the cells were quickly placed together to prevent the electrolyte from evaporating. A lining of Gorilla Epoxy glue was carefully pasted around the edge of the cells where the two half-casings meet. Care was taken to ensure that the glue did not contact the exposed current collector terminals. The cells were then individually wrapped in several layers of Bemis Parafilm “M” laboratory film to hold the cells in place. Paper clamps were used to keep pressure on the cells as the glue was cured overnight. Once clamps were removed, two circular holes were cut on either side of the cells, exposing the conductive current collector terminals for cell analysis.

### Material Characterization

Raman scattering was performed using an Ocean Optics QE65PRO Raman Spectrometer using a 25 mW Ar^+^ laser with 532 nm excitation. The laser beam was focused onto the slurry samples using a 40× objective lens, and spectra were collected using a CCD camera. For each of the slurry batches, two samples were taken and deposited as two distinct “patches” of slurry onto the glass slide. Each batch of slurry is scanned three times, in three different locations, resulting in six Raman spectra altogether for each batch of slurry to study the compositional homogeneity of the mixture.

Scanning electron microscopy (SEM) images were obtained using an FEI Quanta 650 FEG scanning electron microscope (FEI Company, Hillsboro, Oregon, USA), with an average chamber and gun pressure of 1.57 × 10^–4^ mbar and 6.41 × 10^–9^ mbar, respectively. The samples were mounted onto aluminum SEM pin stubs (12 mm diameter, Agar Scientific, Essex, UK) and the chamber was pumped to a high vacuum. A working distance of 10 mm, a spot size of 3.5, and a beam voltage of 10 kV were used in acquiring these SEM images.

### Electrochemical Measurements

Cyclic voltammetry and galvanostatic charge–discharge tests were carried out at room temperature using a BioLogic BCS-805 potentiostat (BioLogic Science Instruments, Seyssinet-Pariset, France). The specific capacitance from the obtained CV curves^50,24^ was calculated using  where *C*_*m*_ is the specific capacitance (F/g), ∫*I dv* is the integrated area of the CV curves (AV), *m* is the mass of electrode active material (g), Δ*V* is the voltage window (V), and *v* is the scan rate (V/s). Specific capacitance was measured from the galvanostatic charge–discharge test data^51^ as , where *C*_*m*_ is specific capacitance (F/g), *I* is applied current (A), *t* is discharge time (s), *m* is mass of electrode active material (g), and Δ*V* is voltage window (V). The specific energy and specific power of the supercapacitor cells were calculated using (52−54) where *E* is specific energy (Wh/kg), *C*_m_ is specific capacitance (F/g), and ΔV is voltage window (V). The specific power of the supercapacitor cells was calculated using  respectively^52−54^ where *P* is specific power (W/kg), *E* is specific energy (Wh/kg), and *t* is discharge time (s). The internal resistance was calculated from ohmic drops present in charge–discharge data under galvanostatic conditions^55^ using  where *R* is total internal resistance (Ω), Δ*V*_*drop*_ is the Ohmic voltage drop (V), and *I* is the applied charging/discharging current (A). The capacitance retention for a cell at a given cycle, relative to the maximum specific capacitance reached by the cell was calculated with  where *C*_*ret*_ is the specific capacitance retention of the cell (%), *C*_*n*_ is the specific capacitance of the cell at a chosen cycle (F/g), and *C*_*max*_ is the maximum specific capacitance achieved by that cell (F/g). The capacity of the supercapacitor cells was calculated with *C = I*Δ*t* where *I* is the discharge current (A), and Δ*t* is the discharge time (s).

## Results and Discussion

### Material Characterization of Chemically Treated FDM-Printed Conductive PLA Current Collectors

**Figure 1:**
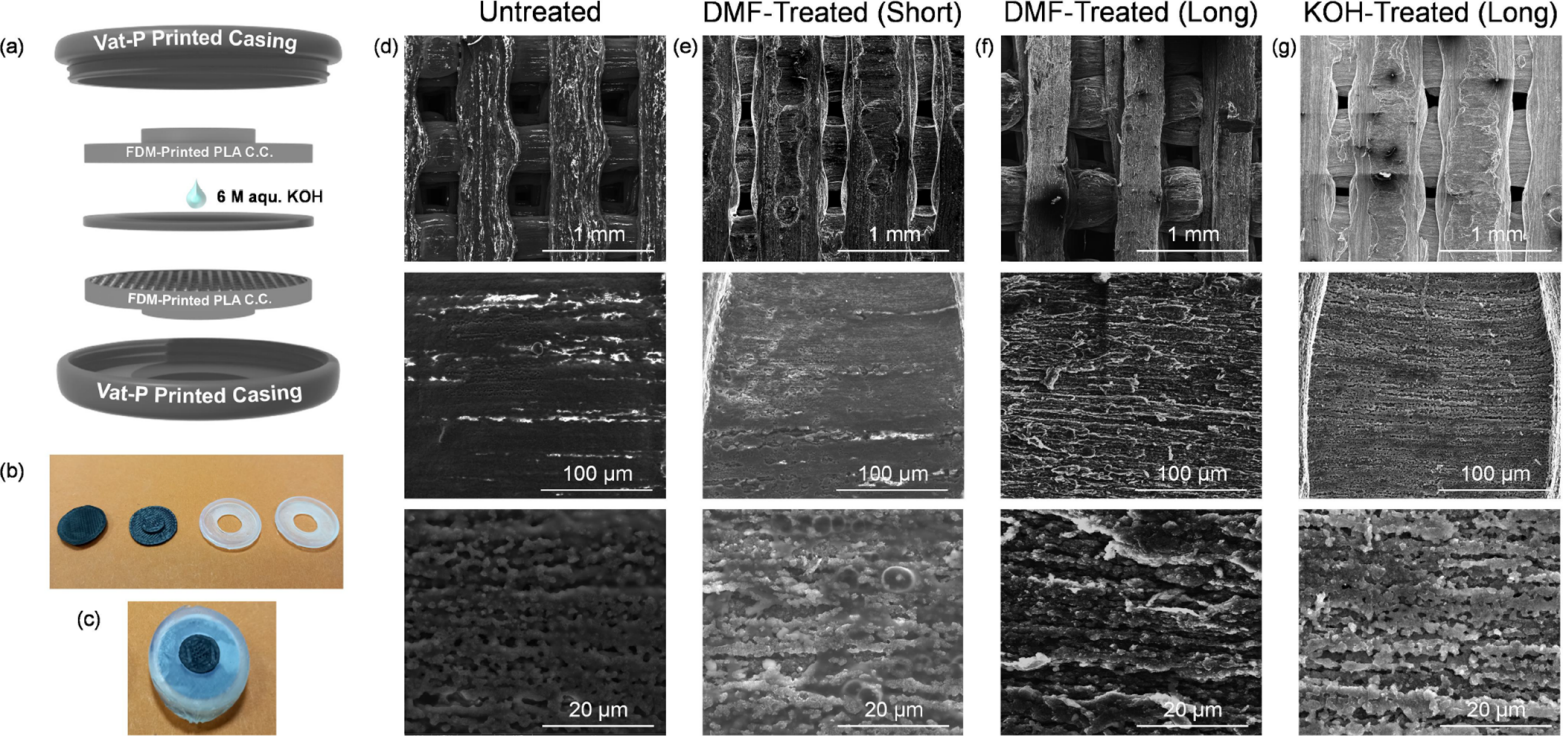
(a) Schematic for the FDM current collector symmetric supercapacitor cell made in this work, consisting of two Vat-P printed outer casings, two conductive PLA FDM-printed current collectors (loaded with carbon electrode slurry after chemical treatment), and a glass microfiber filter acting as a separator soaked in 5 drops of 6 M aqueous KOH electrolyte. Images of (b) FDM-printed current collectors and Vat-P printed outer casings before cell assembly and (c) one fully assembled supercapacitor cell. SEM images at varying magnifications of (d) Untreated unloaded FDM current collector, (e) DMF-treated (Short) unloaded FDM current collector, (f) DMF-treated (long) unloaded FDM current collector, and (g) KOH-treated (long) unloaded FDM current collector.

Figure 1 (a) shows the schematic for the 3D-printed supercapacitor cells studied in this work, consisting of two Vat-P printed outer casings, two FDM-printed conductive-PLA current collectors, and a glass microfiber filter separator soaked in 6 M aqueous KOH electrolyte. The individual 3D-printed components and fully assembled cells can be seen in Figure 1 (b) and Figure 1 (c) respectively. In our previous work with 3D-printed supercapacitor cells,^30^ the FDM-printed conductive PLA current collectors showed poor electronic conductivity when compared to conventional stainless steel current collectors, or to Au-sputtered Vat-P-printed current collectors also studied in that work. This resulted in high internal resistance within the 3D-printed supercapacitor cells, limiting the electrochemical performance of the cells. Simple chemical treatments were proposed as a cheap and straightforward method of improving the electronic conductivity of FDM-printed current collectors. The same cell design is used in this study, as the electrochemical performance issues did not arise from the design or assembly of the supercapacitor cell.

The SEM images in Figure 1 and parts (d–g) show the morphologies of all four types of FDM current collectors used in these experiments after their respective chemical treatments. The filamentary/woodpile structures of the FDM-printed current collectors are clearly visible in the low-magnification images. The Untreated current collector (Figure 1d) has a smoother appearance, with a characteristic texture of smooth nodules in the conductive PLA after thermosetting. The DMF-treated (short) current collector (Figure 1 (e)) shows a rougher morphology, and the DMF-treated (long) and KOH-treated (long) current collectors (Figure 1f, g) show deeper and rougher features and are porous in appearance. The smoother material present in abundance in the Untreated current collector has clearly been removed to varying degrees by chemical treatment methods. The depolymerization at the PLA surface relieves the internal graphitic content in the conductive PLA, and the surface roughness is caused by the random nature of the organic–inorganic composite distribution in the PLA. Excessive decomposition, however, does affect brittleness in these materials.

**Figure 2:**
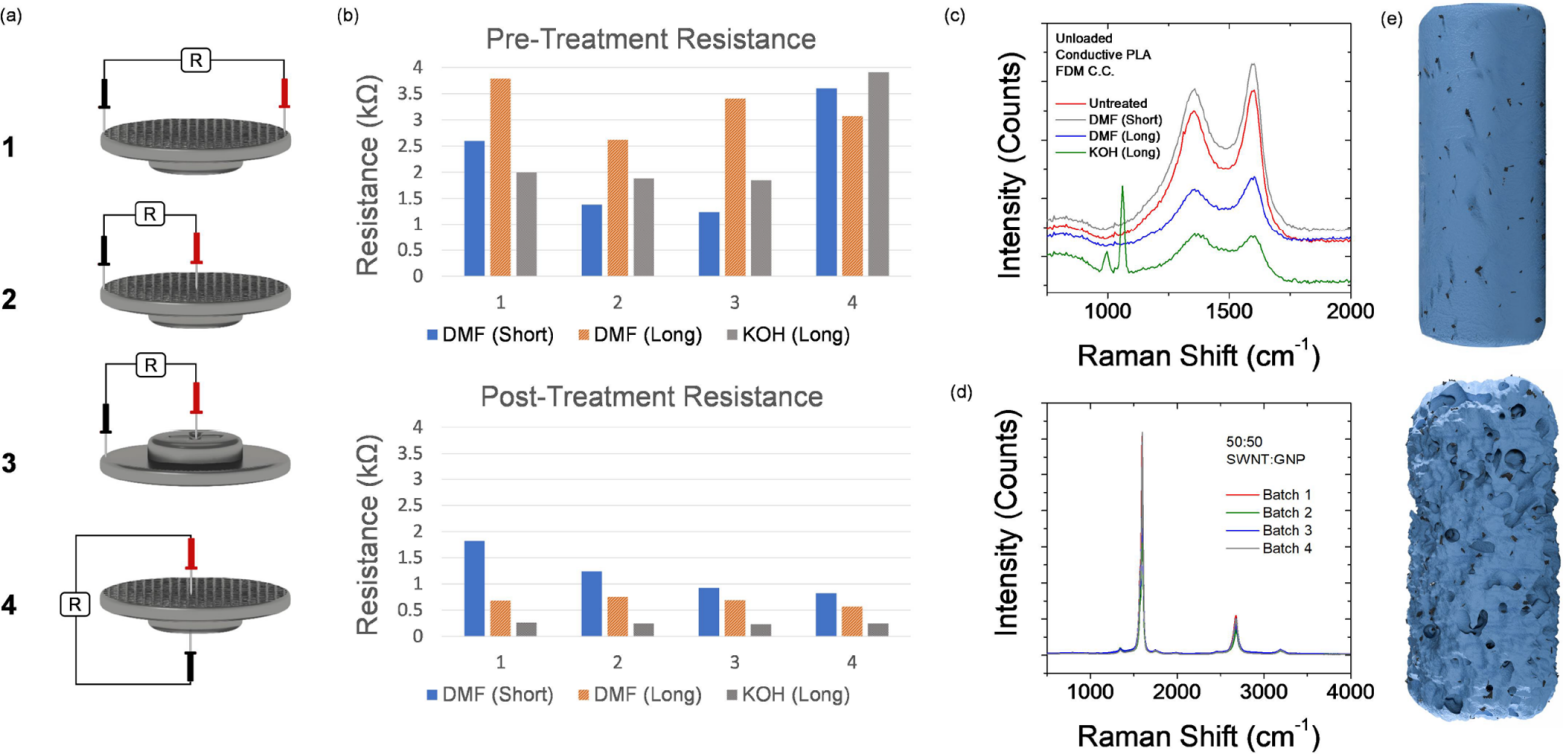
(a) Schematic for resistance measurements taken of the current collectors and the positions of the multimeter probes. (b) Variation of measured resistance before and after chemical treatments. Raman spectra of (c) chemically treated bare FDM current collectors and (d) different batches of 50:50 (SWNT:GNP) slurry used for all cells. (e) Rendered graphics representing a graphite-loaded PLA strand and below it, the modified strand showing increase porosity, surface roughness, and exposure of the near-surface graphitic particles.

Resistance measurements were acquired on the different current collector types pre- and postchemical treatment, according to Figure 2 (a). The resistance data shown in the bar charts in Figure 2 (b) show an obvious reduction in resistance for the chemically treated FDM current collectors, especially for the DMF-treated (long) and KOH-treated (long) current collectors. Correlating with the craggy and porous morphologies seen in the SEM images, the chemical treatment methods removed some of the insulating polymer material from the conductive PLA current collectors. This has the effect of exposing more conductive carbon material and improving the overall conductivity of the FDM current collector interface. Of course, the internal resistance of the FDM is unchanged, and the treatment improves the contact resistance by relieving internal graphitic components at the surface to improve the interfacial conductivity with the slurry and reduce the volume of the resistive polymeric material in the printed material. Both the SEM images and resistance data show that the DMF-Treated (Long) and KOH-Treated (Long) current collectors have been more extensively stripped of their insulating polymer material.

The Raman spectra for all chemically treated current collectors are shown in Figure 2 (c). G-bands (∼1600 cm^–1^) (arising from the stretching of C–C bonds in sp^2^ graphitic materials)^56,57^ and D-bands (∼1350 cm^–1^) (arising from disorder in the sp^2^ carbon structure)^58,59^ are present in all current collector types. The ratio of the G-band to D-band intensities indicates the level of disorder in a graphitic carbon structure. The KOH-treated (Long) current collector is the only one with a more intense D-band than the G-band, suggesting a more disordered graphitic structure than the other current collectors after treatment. The composition of PLA is nominally identical in each case, and the graphitic content is randomly distributed. We rationalize the Raman scattering response by considering that the integrated signal is enhanced from the internal materials in the printed current collectors upon significant removal or surface-bound PLA. The KOH-Treated (Long) PLA is porous and rough and exhibits more of the randomly ordered graphitic structures, but a lower overall vibrational mode intensity due to scattering from the rough and porous surface. A signature of the depth of PLA removal is found in the wavenumber region 950–1100 cm^–1^, where we see a signal consistent with C–CH_3_ and CH_3_ rocking modes. These are not present on the PLA surface of untreated samples, or those pretreated^59^ by different means or shorter times (Figure 2). It has been shown how NaOH can cause de-esterification and roughening of PLA,^60^ and this is likely caused by the K^+^ ions here. The rigidity of the final porous and rough structures is maintained by the graphitic content of the filament.

The Raman spectra of the 50:50 SWNT:GNP slurry used as the electrode active material in the supercapacitor cells are shown in Figure 2 (d). The same G-band (∼1600 cm^–1^) and D-band (∼1350 cm^–1^) are present. The G-band in these spectra is more intense than the D-band for all batches of slurry, suggesting a very ordered graphitic carbon structure with relatively few defects present. For each slurry, Raman scattering spectra obtained in different regions show a homogeneous distribution of materials and consistent modes from the respective nanocarbon after 24 h of mixing.

### Cyclic Voltammetric Response from Treated Printed Supercapacitors

For all electrochemical tests, a voltage window of 0.2–0.7 V was chosen, as our previous work^30^ showed signs of electrolyte degradation when using a voltage window of 0.0–1.0 V.^32^ Narrow working voltage is often a disadvantage of using aqueous electrolytes,^61,62^ but here allows unambiguous testing of the effect of current collector pretreatment without parasitic reaction contributions from electrolysis.

**Figure 3:**
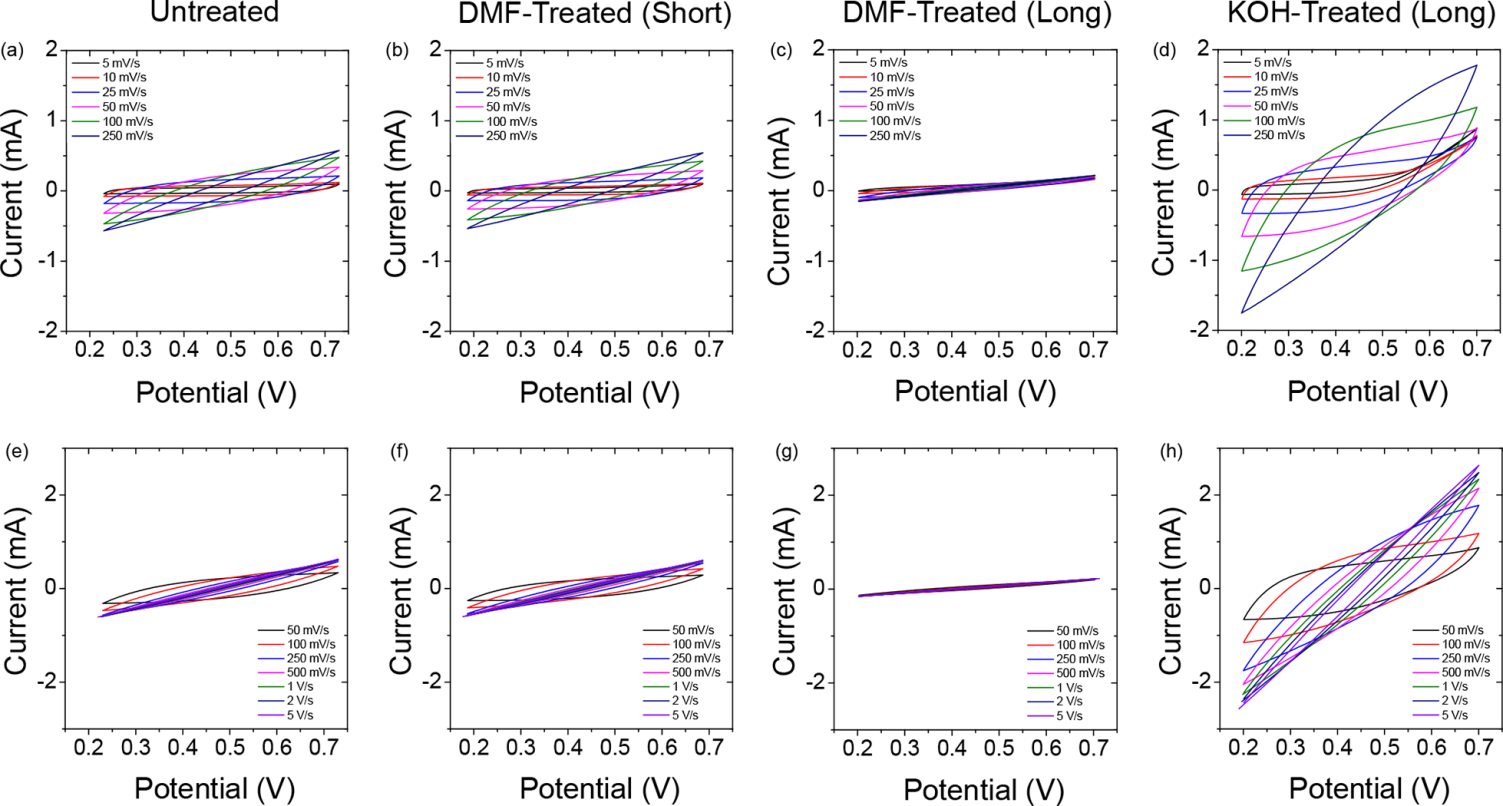
Cyclic voltammetry of conductive PLA FDM current collector printed supercapacitor cells at (a–d) lower scan rates in the range 5–250 mV/s, and (e–h) higher scan rates in the range 50–5,000 mV/s. CVs are shown for (a) and (e) Untreated current collectors, (b, f) DMF-treated (short) current collectors, (c, g) DMF-treated (long) current collectors, and (d, h) KOH-treated (long) current collectors. A voltage window of 0.2–0.7 V was used.

Figure 3 shows the CV responses of the cells studied in this work, at lower scan rates in Figure 3(a-d) ranging from 5 to 250 mV/s, and higher scan rates in Figure 3(e-h) ranging from 50 to 5000 mV/s. Looking at the shapes of the CV curves, it is immediately apparent that all cells suffer from very high internal resistance, as seen by the distinctive, narrow leaf shape of the CV curves,^32,31^ rather than the pseudorectangular shape expected of ideal, highly conductive EDLC behavior.^63,64^ The CV curves presented here lack any anodic/cathodic peaks, suggesting that the energy storage mechanism within these cells is purely an electronic double-layer in nature. However, the high internal resistance of the cells prevents ideal rectangular EDLC responses and hinders the optimal performance of the supercapacitor devices. Table 1 summarizes the integrated charge and capacitance values for each cell calculated from the cyclic voltammetry curves at lower (5 mV/s) and higher (250 mV/s) scan rates.

**Table 1:** Summary of Integrated Specific Charge and Specific Capacitance Values from Cyclic Voltammetry Data As Seen in Figure 3

current collector chemical treatment	lower scan rate (5 mV/s)		higher scan rate (250 mV/s)	
	specific charge (C/g)	specific capacitance (F/g)	specific charge (C/g)	specific capacitance (F/g)
untreated	3.64	7.29	0.14	0.28
DMF-treated (short)	3.34	6.67	0.19	0.37
DMF-treated (long)	1.73	3.46	0.02	0.03
KOH-treated (long)	4.80	9.60	1.05	2.09

Both the untreated (Figure 3a, e) and the DMF-treated (short) (Figure 3b, f) cells show very similar CV responses, both having very narrow, leaf-shaped curves caused by the high internal resistance of the cells. The curves quickly narrow further with increasing scan rate until there is a negligible area within the CV curves. The values in Table 1 also support the similar response of these two cells, although the DMF-treated (short) cell shows a slight improvement over the untreated cell at the higher scan rate (250 mV/s), with ∼1.4× the specific capacitance value of the untreated cell. Compared to all other cells, the DMF-treated (long) cell (Figure 3c, g) performs very poorly. The CV curves of this cell are significantly narrower than the other cells. Looking at the values in Table 1, the DMF-Treated (Long) cell achieves 47% of the specific capacitance of the Untreated cell at 5 mV/s, and only 10% of the specific capacitance of the untreated cell at 250 mV/s.

The KOH-treated (long) cell (Figure 3d and h) shows a different CV response to the other cells. Although still showing the leaf-shaped CV curves indicating high internal resistance, the area within these curves has increased, relative to the Untreated and DMF-treated (short) cells. Table 1 summarizes this improvement in performance. At the lower scan rate of 5 mV/s, the KOH-Treated (Long) cell has 1.3× the specific capacitance value of the untreated cell, while at the higher scan rate of 250 mV/s, it has 7.5× the specific capacitance value of the Untreated cell. Despite this improvement, the KOH-treated (long) cell still shows a CV response very different from the rectangular curves expected of high-conductivity, ideal EDLCs. However, from a plastic supercapacitor cell, the response is nonetheless improved for voltammetric response by electrolyte soaking pretreatment.

### Examining Charge–Discharge Cycle Life Differences in Chemically Treated Printed FDM Supercapacitors

**Figure 4:**
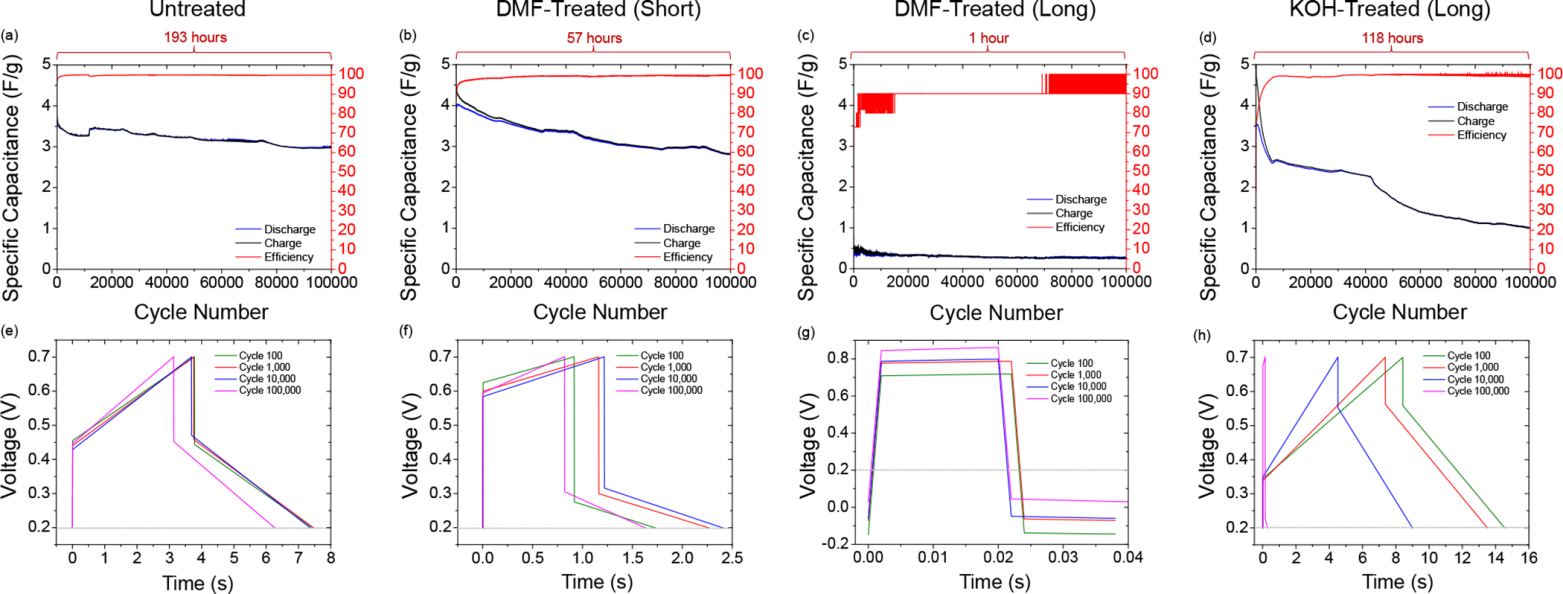
(a–d) Specific capacitance retention of all cell types in this work over 100,000 cycles under galvanostatic conditions. Cells were cycled between 0.2 and 0.7 V at an applied current of ±500 μA (specific applied current of 0.167 A/g). Charge–discharge curves of the cells at different points during the 100,000 cycles under galvanostatic conditions are shown in e–h.

The effect of the chemical treatments on the current collector resistance and interface with the carbon slurry over many charge–discharge cycles under galvanostatic conditions was also investigated. While the capacitance values are obviously limited, the investigation examines how the PLA modification influences the cyclic stability of the charge storage process when the PLA current collectors have improved interfacial conductivity. Figure 4a–d shows the cycle life response for all cells discussed so far, with the specific capacitance values extracted from 100,000 galvanostatic charge–discharge cycles. The untreated (Figure 4a) and DMF-treated (short) (Figure 4b) cells show excellent cycle lives, retaining ∼70% and ∼66% respectively of their maximum specific capacitance values after 100,000 charge–discharge cycles. Both cells also showed excellent and consistent percentage efficiency throughout this test. The cycle lives of these simple 3D-printed symmetric carbon supercapacitors compare well to other research.^65−67^

Following a similar pattern to the CV responses, the DMF-treated (long) cell (Figure 4c) performs very poorly in this test, showing extremely low specific capacitance values and an unsteady percentage efficiency value indicative of unreliable, unstable cell performance. We surmise that DMF, often used to enhance electrospinning and thinning of PLA and other fibers, facilities rearrangement of the polymer with roughening, but without PLA decomposition to the same degree as the depolymerization and de-esterification of PLA by KOH into potassium lactates. The KOH-treated (long) cell (Figure 4d) shows a worsened cycle life compared to the Untreated cell, retaining only ∼30% of specific capacitance relative to the maximum value achieved by the cell. This cell is also more sluggish in reaching its maximum percentage efficiency. The cycle efficiency begins to waver slightly after 60,000 charge–discharge cycles. These results indicate that although the KOH-treated (long) current collector cells showed improved conductivity (as shown in the resistance data and CV responses), this worsened the cycling stability of the cell.

Figure 4e–h shows the charge–discharge curves of the cells at different stages of the 100,000 cycles under galvanostatic conditions. Significant ohmic drops are present in all charge–discharge curves because of the high internal resistance within the cells. The ohmic drop of the Untreated cell (Figure 4e) accounts for approximately half of the voltage window, however, the exceptional cycle life stability of the cell prevents the ohmic drop from worsening. Only a slight reduction in discharge time is observed for this cell after 100,000 charge–discharge cycles. The DMF-treated (short) and DMF-treated (long) cells (Figure 4f,g) show higher ohmic drops than the Untreated cell, despite the evidence for lower current collector resistance shown in the resistance data obtained in Figure 2b. The DMF-treated (short) cell still displays excellent cycle life stability, as the ohmic drop remains very similar after 100,000 charge–discharge cycles, with a slight reduction in charging and discharging time, as reflected in the reduction in specific capacitance.

**Figure 5:**
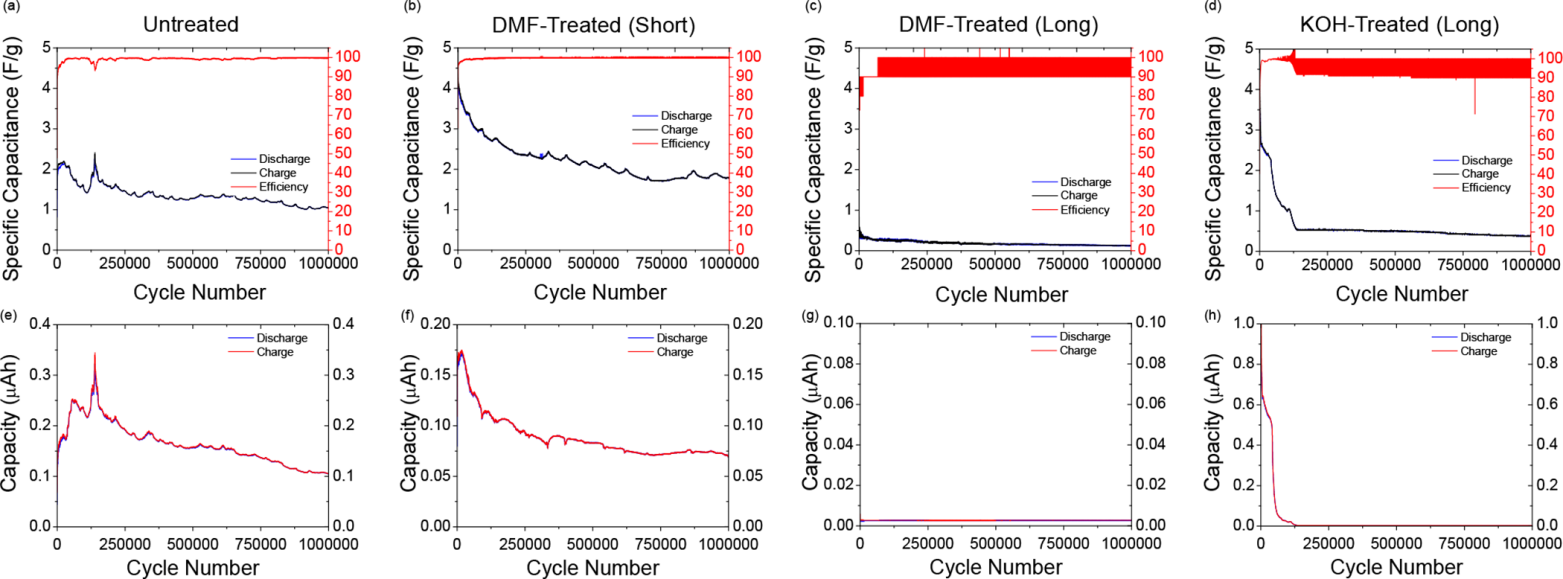
(a–d) Specific capacitance retention of all cell types in this work over 1,000,000 cycles under galvanostatic conditions. Cells were cycled between 0.2 and 0.7 V at an applied current of 500 μA (specific applied current of 0.167 A/g). (e-h) Capacity retention of all cell types derived from specific capacitance data shown in (a–d).

Initially, the KOH-treated (long) cell (Figure 4h) shows a smaller ohmic drop than that of the Untreated cell, reflecting the lower internal resistance of this cell. However, the worsened cycle life stability of the cell is also apparent, as the ohmic drop grows, and the charge and discharge times shrink throughout the charge–discharge cycles. At the 100,000 charge–discharge cycle mark, the ohmic drop has grown to nearly the entire voltage window, and the discharge time to a fraction of the starting value. The internal resistance values calculated from the charge–discharge curves are presented in Table 2. Thus, a failure mode is identified in PLA materials in spite of modification to improve interfacial conductivity which benefits a faster voltammetric response. Under fixed current conditions, the internal volumetric resistance of the PLA and long-term exposure to the electrolyte over many cycles result in continued roughening that severely limits the overall performance (capacity, power) in relatively simple aqueous electrolytes. In Figure 5, we see cell death when the capacitance response becomes obviously noisy in Figure 5c, d, for example, which corresponds with the flooring of the capacitance values even though the resistance measured at the surface has not dramatically changed since its value at 100 cycles.

**Table 2:** Summary of Internal Resistance Values Calculated from Charge–Discharge Curves under Galvanostatic Conditions As Seen in Figure 4

current collector chemical treatment	resistance at cycle 100 (Ω)	resistance at cycle 100,000 (Ω)
untreated	257	249
DMF-treated (short)	425	396
KOH-treated (long)	141	474

Figure 5 a–d shows the cycle lives of all cells across 1,000,000 charge–discharge cycles under galvanostatic conditions. The same pattern seen in the 100,000 charge–discharge cycles continues here, as both the Untreated and DMF-Treated (Short) cells (Figure 5a,b) show excellent cycling stability, retaining ∼47 and ∼42% respectively of their maximum specific capacitance values after 1,000,000 charge–discharge cycles. The percentage efficiency of these cells remains steadily around 100%, with very little oscillation. The KOH-Treated (Long) cell again shows a worsened cycle-life stability, retaining ∼10% of its maximum specific capacitance as well as an unsteady percentage efficiency for most of the test. Figure 5e–h shows the cycle lives of the cells in terms of capacity across 1,000,000 charge–discharge cycles under galvanostatic conditions. Table 3 shows the percentage capacity retentions of the cells at different stages in the charge–discharge tests. The high cycle life stability of the Untreated and DMF-treated (short) cells and the relatively low cycle life stability of the KOH-treated (long) cells are very apparent from these figures and percentage values. It is quite possible that the conductive PLA current collectors, already extensively etched by the long KOH treatment, begin to lose their structural integrity upon further (even longer) exposure to the 6 M aqueous KOH electrolyte, resulting in serious long-term stability problems seen in these results.

**Table 3:** Maximum Capacity Values Achieved by Each Cell and Capacity Values at Different Stages of the 1,000,000 Charge–Discharge Cycles in Terms of Percentages of Maximum Capacity

current collector chemical treatment	maximum capacity (μAh)	percentage of maximum capacity		
		cycle 10,000	cycle 100,000	cycle 1,000,000
untreated	0.323	50%	70%	32%
DMF-treated (short)	0.172	96%	66%	40%
DMF-treated (long)	0.00278	90%	100%	90%
KOH-treated (long)	0.871	72%	2%	0.3%

### Examining Rate Response Differences in Chemically Treated Printed FDM Supercapacitors

**Figure 6:**
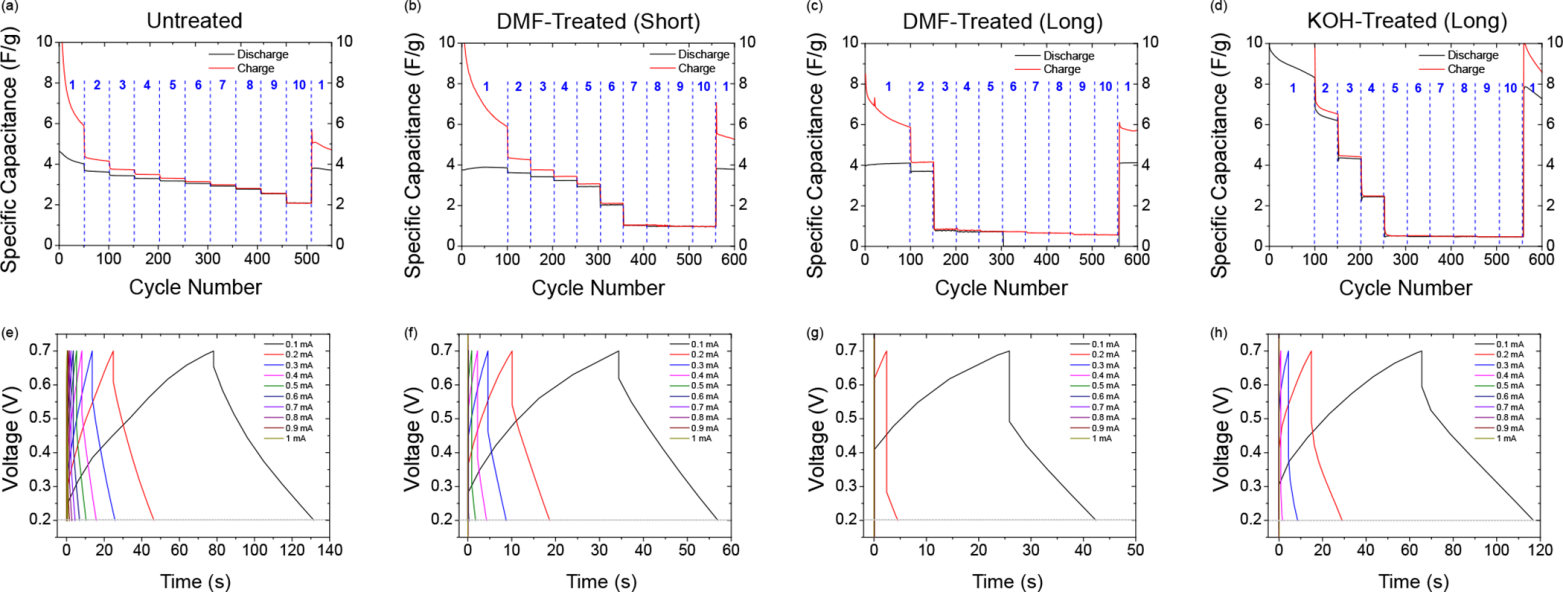
(a–d) Varied-current life-cycle plot for all cells studied in this work under galvanostatic conditions with an increasing applied current. The blue numbers between the dotted lines correspond to the charging and discharging current used in that cycle period, progressing through 1 (±100 μA or 0.033 A/g), 2 (±200 μA or 0.067 A/g), 3 (±300 μA or 0.100 A/g), 4 (±400 μA or 0.133 A/g), 5 (±500 μA or 0.167 A/g), 6 (±600 μA or 0.200 A/g), 7 (±700 μA or 0.233 A/g), 8 (±800 μA or 0.267 A/g), 9 (±900 μA or 0.300 A/g), and 10 (±1 mA or 0.333 A/g) then returning to 1 (±100 μA or 0.033 A/g). Cells were cycled between 0.2 and 0.7 V. (e–h) Charge–discharge curves of the cells at each of the increasing applied currents.

Rate capability tests were carried out on all cells to examine the influence of current collector chemical treatments on the stability of their interface with the active materials and also to investigate the impact of PLA failure after longer exposure to electrolytes at a fixed current. Figure 6 shows the responses of all cells extracted from galvanostatic charge–discharge tests. The untreated current collector FDM-printed supercapacitor cell best retains its specific capacitance over repeated cycling at successively higher rates (52% retention after a 10-fold increase in applied current, 100 μA to 1 mA). The chemically treated current collector FDM-printed supercapacitor cells perform worse, retaining only 25% (DMF-treated (short)), 0% (DMF-treated (long)), and 6% (KOH-treated (long)) of their specific capacitance after a 10-fold increase in applied current. All four cells recover almost all specific capacitance (93–99%) after returning to the initial rate.

The results of these varied-current galvanostatic tests show that all the FDM current collector cells have poor rate capability and are not suited for high current density applications but, with modification, are potentially useful for low power, small cell applications where the printable materials can be readily recycled. The low capacitance retention can be attributed to the high resistance of the FDM current collectors, resulting in inefficient charge transfer during the discharge processes at higher currents. In fact, the chemical treatment methods have worsened the rate capability of the cells when compared to the Untreated cell performance, despite initially improving the capacitance. Under voltammetric conditions, the treatment shows marked improvement in response, showing that the interfacial conductivity improvement of the PLA is potentially dependent, requiring lower currents linked to the resistance change. Though comparing well to other studies in terms of cycle life, the cells presented in this work lack the good rate capability present with other carbon nanotube/graphene-based supercapacitors which utilize more conductive current collector components in their devices^68−71^ but is instructive for understanding the nature of PLA and FDM cell components for aqueous energy storage systems.

For all cells tested in this work, their specific capacitance values and discharge times from Figure 6 were used to calculate their specific energies and specific powers applied currents of 0.1, 0.5, and 1 mA which are outlined in Table 4. Due to poor rate performance at higher applied currents, the specific energies of the treated cells at 1 mA were unobtainable (because of the distorted voltage window stretching beyond the voltage window in the settings, and minuscule discharge times).

**Table 4:** Specific Energies and Specific Powers were Calculated from the Galvanostatic Rate Capability Tests

current collector chemical treatment	specific energy of electrode material (Wh/kg)			specific power of electrode material (W/kg)		
	at 0.1 mA	at 0.5 mA	at 1 mA	at 0.1 mA	at 0.5 mA	at 1 mA
untreated	0.12	0.03	0.0005	7.76	23.50	6.57
DMF-treated (short)	0.09	0.004		15.04	18.54	
DMF-treated (long)	0.05			10.58		
KOH-treated (long)	0.18	0.000025		12.67	3.75	

At the currents applied in this work, the specific energy and power values are very low for EDLC devices. Specific energies of 1–10 Wh/kg and specific powers of 500–10,000 W/kg are typical for EDLC devices.^72−75^ The specific energies (therefore, specific powers) of the devices in this study suffer from the narrow operating voltage window and the resistance values that remain too high for mA-level galvanostatic cycling. Potentiodynamic responses, useful not only for some supercapacitor applications but also in electrochemical sensors, for example, are much better due to electrolyte presoaking.

## Conclusions

For hydroxide-based electrolytes, presoaking was shown to improve the interfacial conductivity of conductive additive-impregnated 3D-printed PLA current collectors, compared to chemical decomposition or modification using DMF. By depolymerizing the outer surface, exposed graphite improves the interfacial conductivity with the carbon-based active materials in the symmetric supercapacitor. These FDM-printed current collectors designed to fit with a Vat-P printed cell show a moderate electrical conductivity increase for the DMF-treated (short) current collectors and a significant reduction in resistance for the DMF-treated (long) and KOH-treated (long) current collectors. SEM images of the current collector morphology confirm the removal of nonconductive material from the conductive PLA current collectors via electrolyte presoaking. Cyclic voltammetry tests show near-identical responses from the untreated and DMF-treated (short) cells and an improved response for the KOH-treated (long) cell, which is useful for some voltammetric charge storage applications or electrochemical sensors where the fast response is dominated by the surface activity with current values linked to the driving voltage. Galvanostatic data, however, at higher fixed current shows stable and incrementally improved capacitance, but the higher surface conductivity PLA after long KOH presoaking showed a worse cycle response compared to untreated FDM.

We identified a failure mode for PLA linked to decomposition that fundamentally limits its use in systems such as these with relatively simple aqueous electrolytes over longer durations. This results in a trade-off between improved specific capacitance from exposed active graphitic materials and greater surface area and less robust PLA composite that degrades over long cycling durations. The limitation in long cycle life efficiency is fundamental to PLA current collectors and electrodes in such 3D printed aqueous supercapacitors, and future work should consider the impact of these types of electrolytes on printed passive or active materials in energy storage devices. For electrochemical sensors using printed electrodes, voltammetric responses can be improved with surface interfacial conductivity and area modification, but a galvanostatic response over long durations is much less performant. In a system using organic electrolytes, the printed composite matrix will be important. Balancing ductility in the printed current collector with (often more brittle) higher active material concentration requires further research to ensure better performance and more durable material.
